# 2567. Clinical Outcomes of Vancomycin Area-Under-the-Curve Monitoring: A Quasi-Experimental Study

**DOI:** 10.1093/ofid/ofad500.2184

**Published:** 2023-11-27

**Authors:** Alina Viteri, Natalie Tucker, Ryan Flynn, Jena K Foreman

**Affiliations:** HSHS St. John's Hospital, Springfield, Illinois; HSHS St. John's Hospital, Springfield, Illinois; Monument Health, Rapid City, South Dakota; HSHS St. Elizabeth's Hospital, O'Fallon, Illinois

## Abstract

**Background:**

Previous studies that demonstrated decreased nephrotoxicity with area-under-the-curve (AUC) vancomycin dosing compared to trough monitoring used two levels and a spreadsheet model for calculating AUC. The purpose of this study was to evaluate if the same reduction in acute kidney injury (AKI) was achieved while using a single level Bayesian monitoring program.

**Methods:**

This retrospective quasi-experiment was performed at two sites within the same health system. The primary objective was to assess the rate of AKI between vancomycin dosed by trough monitoring as compared to AUC monitoring using a Bayesian software program. Secondary objectives included mean daily dose of vancomycin, number of vancomycin levels, and severity of AKI. Patients were included in the study if they were 18 years or older with at least one vancomycin concentration drawn. Patients were excluded if they had a baseline serum creatine greater than 2 mg/dL, received renal replacement therapy of any type, or received > 1 dose of vancomycin prior to admission.

Patients were matched 1:1 based on acute physiology and chronic health evaluation II score, body mass index, and vancomycin indication. Multivariable logistic and Cox proportional hazards regression were used to examine the independent association between the monitoring strategy (trough vs AUC) and nephrotoxicity.

**Results:**

Few differences in baseline characteristics were identified between groups. More patients were treated for pneumonia, bacteremia, or urinary tract infection in the trough group, whereas more were treated for skin and soft tissue infection in the AUC group. The AUC group (255 patients) had a 16% incidence of AKI in comparison to the trough group (265 patients) with a 19% incidence of AKI (p = 0.517). Of the secondary outcomes, those in the AUC group received lower daily doses than patients in the trough group (p = 0.015). Multivariable regression analysis identified similar results for each of the study outcomes including the primary outcome (OR 1; 95% CI 0.62 – 1.63).

**Conclusion:**

The use of a single level Bayesian monitoring for vancomycin dosing did not result in a decreased incidence of acute kidney injury when compared to trough monitoring.

**Disclosures:**

**All Authors**: No reported disclosures

